# Psychotropic prescribing practices in adults with intellectual disability and autism spectrum disorder in Richmond Neurodevelpmental Services

**DOI:** 10.1192/bjo.2021.242

**Published:** 2021-06-18

**Authors:** Anushka Dissanayake, Nicholas Davey, Rupal Patel

**Affiliations:** 1 Your Healthcare (Neurodevelopmental Services); 2 South West London St Georges Mental Health Trust

## Abstract

**Aims:**

Our aim was to evaluate psychotropic prescribing practices in adults with intellectual disability (ID) and autism spectrum disorder (ASD) across the Richmond Neurodevelopmental Service (NDS).

Stopping over-medication of people with a learning disability, autism or both with psychotropics (STOMP) aims to reduce the potential harm of inappropriate use of psychotropic medications. We aimed to evaluate our prescribing practices in keeping with STOMP and the NICE guidelines.

**Method:**

We collected information from our clinical records on patients that met the inclusion criteria (≥18 years + diagnosis of ID and autism) from October-November 2019. We gathered the following: age, sex, severity of ID, psychiatric diagnoses, psychotropic medication, presence of challenging behaviours, involvement of positive behaviour support (PBS) and documentation of a PBS plan.

**Result:**

32 patients met our criteria (3:1 Male-Female ratio with an age range of 20-74 (Median 33 years old)). All 32 patients showed evidence of challenging behaviours. In the cohort, mild ID represented 18.8% (n = 6), moderate ID 40.6% (n = 13) and severe ID 40.6% (n = 13).

17 patients (53%) had a PBS plan in place. For those without a PBS plan (47%, n = 15), a referral to behavioural analysis had been considered/requested in 67% (n = 10).

31 patients were on psychotropic medication and 84% (n = 26) had an indication documented in the notes although every patient had had a medication review in the last 6 months. 67.7% (n = 21) of the prescriptions were for challenging behaviours.

The average number of medications prescribed was 2 (median 2, mean 2.41) but this was reduced to 1 (median 1, mean 1.76) when additional psychiatric diagnoses and epilepsy were excluded.

**Conclusion:**

Prescriptions are regularly reviewed in keeping with STOMP guidance but there is more scope for utilising behaviour analysis input as well as the need to improve documentation of the rationale for psychotropic medications.

